# A Pathological Assessment of the Microvasculature of Biliary Tract Neoplasms Referring to Pre-Existing Blood Vessels and Vessel Co-Option

**DOI:** 10.3390/cancers16223869

**Published:** 2024-11-19

**Authors:** Yasuni Nakanuma, Zihan Li, Yasunori Sato, Motoko Sasaki, Kenichi Harada, Yuko Kakuda, Takashi Sugino

**Affiliations:** 1 Division of Pathology, Shizuoka Cancer Center, Shizuoka 411-8777, Japan; y.kakuda@scchr.jp (Y.K.); t.sugino@scchr.jp (T.S.); 2 Department of Diagnostic Pathology, Fukui Prefecture Saiseikai Hospital, Fukui 918-8503, Japan; 3 Department of Human Pathology, University Graduate School of Medicine, Kanazawa 920-8640, Japan; lizihan19960823@hotmail.com (Z.L.); sato-ya@med.kanazawa-u.ac.jp (Y.S.); m8sasaki@med.kanazawa-u.ac.jp (M.S.); kenichih@med.m.kanazawa-u.ac.jp (K.H.)

**Keywords:** vessel co-option, neoangiogenesis, biliary tract, carcinoma and precursors, invasion, structure–function relationship

## Abstract

There are several types of microvasculature supplying neoplasms: “newly formed blood vessels” (neoangiogenesis), which are a component of the tumor microenvironment (TME) of invasive carcinoma with wound healing-like reaction; and “pre-existing blood vessels”, which are used as tumor-supplying vessels by neoplasms (co-option vessels) and are likely to develop in hypervascularized organs. We herein review the microvasculature of neoplasms of biliary tract with reference to pre-existing vessels and vessel co-options. In the hepatobiliary system, intrahepatic large and extrahepatic bile ducts (large bile ducts) and the gallbladder as well as hepatic lobules are highly vascularized regions. In large bile ducts, the biliary lining epithelia and underlining capillaries (peribiliary capillary plexus [PCP]) form the biliary epithelia–PCP alignment, whereas the hepatocyte–sinusoid alignment composes hepatic lobules. Cholangiocarcinoma (CCA) and gallbladder carcinoma (GBC) are the main biliary tract carcinomas. CCA is subdivided into distal (d/CCA), perihilar (pCCA), and intrahepatic (iCCA), and iCCA is subdivided into small duct type (SD-iCCA) and large duct type (LD-iCCA). High-grade biliary intraepithelial neoplasm (BilIN), intraductal papillary neoplasm of the bile duct (IPNB), pyloric gland adenoma (PGA), and intracholecystic papillary neoplasm (ICPN) have recently been proposed as the precursors of LD-iCCA, p/dCCA, and GBC. In the large bile ducts and gallbladder, all cases of high-grade BilIN and PGA, about half of IPNB, and one-third of ICPN with less-complicated structure were found to have hijacked the PCP as their supporting vessels (vessel co-option), while p/dCCA, LD-iCCA, and GBC were supplied by neo-angiogenetic vessels associated with fibrous stroma. The intraluminal components of the remaining cases of ICPN and IPNB with complicated structure presented sparse capillaries without fibrous stroma, a unique microvasculature different from that of co-option or neoangiogenesis. Regarding iCCA showing invasion into the hepatic lobules, some SD-iCCAs replaced hepatocytic cords and used pre-existing sinusoids as co-opted vessels. Visualization of pre-existing vessels could be a new pathological tool for the evaluation of malignant progression and of vascular supply in CCAs and its precursors.

## Simple Summary

In large bile ducts, the biliary lining epithelia and underlining capillaries (peribiliary capillary plexus [PCP]) form the biliary epithelia–PCP alignment, whereas the hepatocyte–sinusoid alignment composes hepatic lobules. High-grade biliary intraepithelial neoplasm (BilIN) and intraductal papillary neoplasm (IPNB), and intracholecystic papillary neoplasm (ICPN) have recently been proposed as the precursors of biliary tract carcinoma (BTC). All cases of high-grade BilIN and PGA, about half of IPNB, and one-third of ICPN with less-complicated structure were found to have hijacked the PCP as their supporting vessels (vessel co-option), while BTC were supplied by neo-angiogenetic vessels associated with fibrous stroma. The intraluminal components of the remaining cases of ICPN and IPNB with complicated structure presented sparse capillaries without fibrous stroma. Some BTC replaced hepatocytic cords and used pre-existing sinusoids as co-opted vessels. Visualization of pre-existing vessels could be a new pathological tool for the evaluation of vascular supply in BTC and its precursors.

## 1. Introduction

The vascular supply of neoplasms is diverse and unique to the individual neoplasms and organs from which the neoplasms arise [1,2,3,4,5,6,7]. Neoplasms can also change the tumoral blood supply along with malignant progression. Several types of tumor-supplying vessels have been reported thus far [1,2,3,4,5,6,7]: newly formed blood vessels (neo-angiogenesis) in the tumor fibrotic microenvironment (TME) of invasive carcinoma and pre-existing blood vessels that are used as supporting vessels by neoplasms (vessels co-option) [1,6]. Neo-angiogenesis is a dominant entity of the TME of invasive carcinoma with a wound healing-like reaction, in addition to extracellular matrix (ECM) or fibrosis and cancer-associated fibroblasts (CAFs) [1,2,3,4,5]. It has been the focus of oncology research in the past, linking tumor-associated angiogenesis with the progression of carcinoma and anti-angiogenic agents for carcinoma treatment [1,5]. In contrast, vessel co-option is a non-angiogenic mode in which tumor cells hijack pre-existing blood vessels of the organs to supply blood and nutrients to the tumor, and this blood supply mechanism is most commonly found in primary and metastatic tumors in highly vascularized organs, such as the lung, brain, and liver [1,3].

Biliary tract carcinomas can be classified into intrahepatic CCA (iCCA), perihilar CCA (pCCA), distal CCA (dCCA), and gallbladder carcinoma (GBC) [8,9]. iCCA is further divided into the small duct type (SD-iCCA) and large-duct type (LD-iCCA). Recently, several pre-invasive precursors, such as high-grade biliary intraepithelial neoplasm (BilIN), intraductal papillary neoplasm of the bile duct (IPNB), intracholecystic papillary neoplasm (ICPN), and pyloric gland adenoma (PGA), have also been proposed in the large bile ducts and gallbladder [8,9]. However, studies on the microvasculature of these biliary tract neoplasms referring to vessel co-option have been very limited thus far [10,11,12].

In the hepatobiliary system, there are physiologically two anatomical regions showing hypervascularization (Table 1): (i) intrahepatic large and extrahepatic bile ducts (large bile ducts) and the gallbladder and (ii) hepatic lobules [11,12,13,14]. We recently reported that the peribiliary capillary plexus (PCP), a component of the peribiliary vascular plexus (PVP) of the biliary tract, may be used as a vessel co-option in biliary tract neoplasms at early stages [11,12]. Metastatic adenocarcinomas and hepatocellular carcinoma (HCC) have also been reported to replace growth in hepatic lobules and use sinusoid vessels as co-opted vessels [1,15]. Recently, we also reported that hepatic sinusoid vessels are used as co-option vessels in SD-iCCA showing the replacing growth pattern to the hepatic parenchyma [11]. The pathologic assessment of the microvasculature of biliary tract neoplasms and their vascular supply may lead to the clarification of their pathogenesis and the progression from precursors to invasive carcinoma, as well as to the therapeutic developments, such as anti-angiogenic therapy, for these neoplasms [1].

This review summarizes the observation from our previous studies with further studies in the literature to give an overview of (i) the microvasculature of LD-iCCA, p/dCCA, GBC, and their precursors referring to pre-existing vessels of large bile ducts and gallbladder, such as PCP, and vessel co-option, and of (ii) the growth of iCCA at the interface between the tumor and hepatic parenchyma, referring to the hepatic sinusoids and vessel co-option. We highlight the fact that visualization of pre-existing blood vessels and vessel co-option opens a new field in the pathology of biliary tract neoplasms. In this review, vascular endothelia were mainly evaluated by immunostaining for CD34, CD31, collagen type IV, and factor VIII in addition to routine histologic examinations.

## 2. Microvasculature of Neoplasms Arising in Large Bile Ducts and Gallbladder Referring to Pre-Existing Vessels and Vessel Co-Option

### 2.1. Non-Neoplastic Pattern of Microvasculature

**Figure 1:**
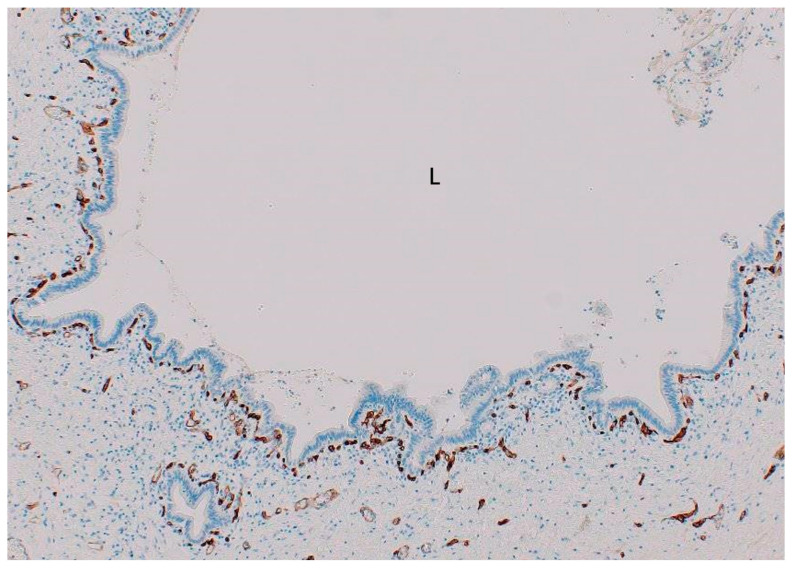
Dotted capillaries are located just beneath of the lining biliary epithelia (peribiliary capillary plexus [PCP]) in non-neoplastic intrahepatic large bile duct. L, bile duct lumen. CD34 immunostaining.

The biliary tract is supplied by the PVP [3,13]. Just under the lining epithelia of the large bile ducts and the gallbladder lies a single layer of densely and regularly dotted capillaries (with a chain-like pattern) composed of similar diameter with a typical round mesh structure called the PCP [11,12] (Figure 1), which stems from hepatic arterial branches [3,11,12]. Venous blood from the PCP flows into the portal vein branches or hepatic sinusoids. The PCP is continuously distributed densely and regularly, forming a capillary network, at the large bile ducts and gallbladder (biliary epithelia–PCP alignment) (Table 1) [3,11,12], which is also identifiable around and within the peribiliary glands and their conduits in the bile duct wall as well as in non-neoplastic glands of the gallbladder wall, including the Rokitansky–Aschoff sinus (RAS) [11,12]. However, the PCP becomes sparse and irregular in smaller bile ducts, particularly the interlobular bile ducts and bile ductules [3,11,12]. The PCP is a component of the lamina propria of the biliary tract mucosa and tightly adheres to the biliary lining epithelia [11,12]. Ultrastructurally, the PCP is composed of fenestrated endothelium with a diaphragm and extreme cytoplasmic attenuation [16], suggesting that it might be arranged to transport substances effectively by way of biliary epithelial cells and that the biliary epithelia–PCP structure is significantly involved in the function of the biliary tract, such as active secretion and absorption of bile constituents like bile acid, water, electrolytes, and other constituents from biliary epithelia into the bile and vice versa [11,12,16].

Pathologically, in the regenerative and hyperplastic processes of gallbladders and large bile ducts after various injuries, a single layer of the PCP is structurally retained just beneath the lining epithelia [11,12]. For example, in extrahepatic biliary obstruction, the PCP increases around proliferated lining epithelia and peribiliary glands [3,17]. Biliary epithelial cells are reportedly involved in PVP proliferation and neoangiogenesis [3,17]. That is, proliferating biliary epithelial cells are an important source of vascular endothelial growth factor (VEGF), which may play a role in driving PVP proliferation around the biliary epithelia [3,17]. In contrast, during hepatic arterial ligation-induced ischemia of the biliary system, the PVP collapses, and the biliary epithelia show increased apoptosis and decreased VEGF secretion [3,17], suggesting a close link between biliary epithelial proliferation and secretion of VEGF from biliary epithelial cells and proliferation of PVP. This supports the potential role of biliary epithelia in angiogenesis in non-neoplastic and neoplastic cholangiopathy, including CCA and GBC and their precursors [17,18].

### 2.2. Neoplastic Pattern Microvasculature

There are several types of microvasculature supplying LD-iCCA, p/dCCA, GBC, and their precursors (Table 2) [10,11,12].

#### 2.2.1. Microvasculature of Invasive LD-iCCA, p/dCCA, and GBC

**Figure 2:**
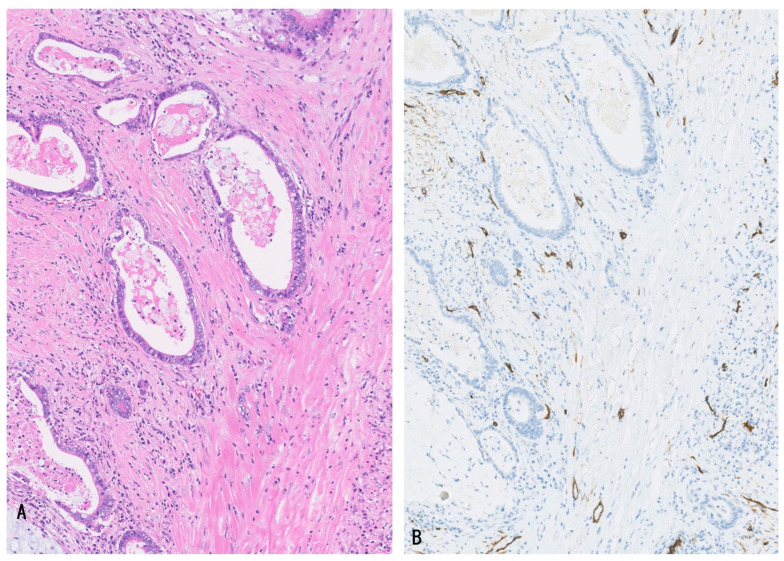
In the fibrous stroma of invasive gallbladder carcinoma, several vessels positive for CD34 are found. They are not oriented to carcinoma glands. (**A**), HE; and (**B**), CD34 immunostaining.

These invasive carcinomas showed tubular/glandular or solid/cord-like adenocarcinomas embedded within the fibrous stroma, in which small numbers of venous and arterial vessels and capillaries were found. These vessels did not orient toward or surround the invasive tubular/cord-like carcinoma (Table 3) (Figure 2A,B). These vessels correspond to neo-angiogenesis in the fibrotic stroma of invasive carcinoma, with a wound healing-like reaction with fibrosis and inflammatory cells [1,11,12]. Such a tumor-supportive microenvironment with neoangiogenesis is well-known in pancreatic duct adenocarcinoma (PDAC) [5], a counterpart of LD-iCCA, p/dCCA, and GBC [19], and such microenvironment in PDAC is reportedly hypovascular and important for the aggressive and invasive characteristics of the TME of PDAC [5]. These characteristics of PDAC microenvironments could be the case in p/dCCA, LD-iCCA, and GBC [19].

#### 2.2.2. Microvasculature of Precursors (High-Grade BilIN and IPNB, ICPN, and PGA)

##### High-Grade BilIN

This lesion is a microscopically identifiable, intraepithelial pre-invasive biliary epithelial neoplasm and presents with flat, pseudopapillary, or micropapillary pattern with hyperchromatic nuclei and disordered polarity [8,9]. High-grade BilIN involves the mucosa of the large bile ducts and gallbladder to a considerable extent and does not infrequently involve the peribiliary glands of the bile ducts and the Rokitansky–Aschoff Sinus (RAS) of the gallbladder [8,9]. High-grade BilIN is an important precursor of LD-iCCA, p/dCCA, and GBC [8,9], presenting an abrupt transition to the surrounding nonneoplastic biliary epithelia.

**Figure 3:**
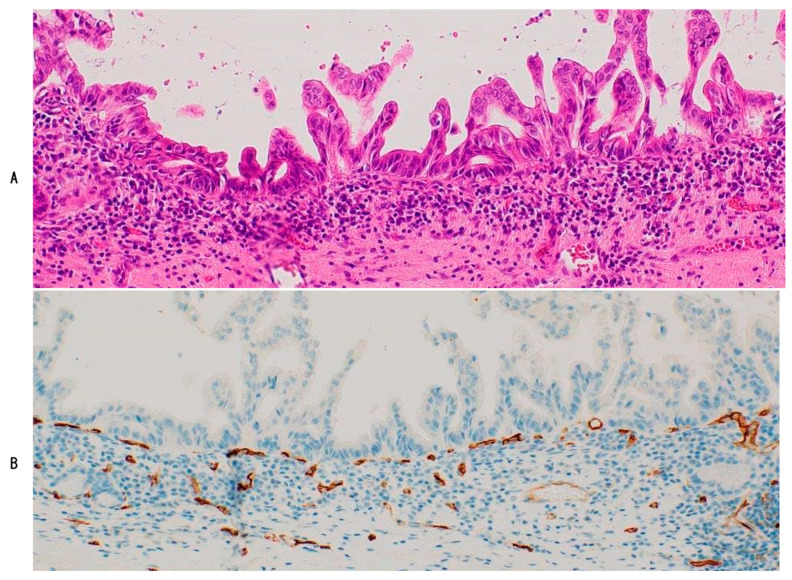
Dotted chain-like capillaries resembling peribiliary capillary complex (PC) of normal bile ducts are located under neoplastic epithelia of high-grade biliary intraepithelial neoplasm (BilIN). (**A**), HE and (**B**), CD34 immunostaining.

The epithelial lining of high-grade BilIN is always underlined by regular and dense dotted capillaries (PCP-like capillaries) (Table 3) (Figure 3) [11,12]. These PCP-like capillaries are continuous with PCP in the surrounding non-neoplastic biliary mucosa, suggesting that (i) the non-neoplastic epithelia of the bile duct and gallbladder were intraepithelially replaced by the neoplastic epithelia of high-grade BilIN, and (ii) pre-existing PCP of these non-neoplastic lining epithelia might have been hijacked and used as supplying vessels of high-grade BilIN (vessel co-option) [1,11,12]. High-grade BilIN seems to require a blood supply for growth, similar to non-neoplastic biliary epithelia. These processes resemble the regenerative process of biliary lining epithelia, which finally restores the biliary tract [11]. The mechanisms involved in the regeneration and replacement of biliary lining epithelia after injuries may also be involved in the replacement process of high-grade BilIN.

##### IPNB, ICPN and PGA

Pathological features of IPNB, ICPN, and PGN are briefly described, followed by their neoplastic microvasculatures.

IPNBs are grossly visible preinvasive neoplasms arising in the large bile ducts and are typically >5 mm in height [8,9]. These neoplasms histologically show papillary/villous/tubular epithelial neoplasms with fine stalks and/or narrow stroma. The surrounding bile duct epithelia of these grossly visible intraluminal components also shows flat or micropapillary intraepithelial neoplasms. To further analyze the clinical and pathological features of IPNB, type 1 and 2 subclassifications of IPNB were recently proposed to subgroup IPNB [8,9,20]. The following “irregular and complicated lesions (ICLs)” were found in the intraluminal neoplastic components of IPNB; such cases were classified as type 2. When these ICLs were not found, the cases were classified as type 1. While type 2 showed more aggressive pathologic features, type 1 showing a favorable postoperative survival compared with type 2 [9,21,22].

ICLs include the following: (1) An irregular and complicated cytoarchitecture composed of striking atypical epithelia with hyperchromatic and atypical nuclei; cribriform, solid, and densely packed tubular lesions; and predominant pseudopapillary, serrated, stratified, and cystic lesions; (2) bizarre epithelial cells and nuclei corresponding to overt malignancy; and (3) coagulative necrosis, particularly comedo-like necrosis or intraglandular necrotic debris.

ICPNs are a grossly visible preinvasive neoplasm that predominantly grows in the lumen of the gallbladder. These neoplasms histologically show papillary/villous/tubular neoplasms with fine stalks or narrow stroma, lined by neoplastic epithelia. The surrounding mucosa also show flat or micropapillary intraepithelial neoplastic spread as seen in IPNB [9]. These flat or micropapillary neoplastic lesions show an abrupt or gradual transition to the non-neoplastic surrounding biliary mucosa. As with IPNB, ICPNs are also subclassified into types 1 and 2 [9].

PGAs are a sizable, pedunculated polypoid lesion of the gallbladder composed of tubular or acinar neoplastic components resembling gastric pyloric glands, histologically and immunohistochemically, with thin stalks or narrow stroma [8]. While PGA usually presents with low-grade dysplasia, high-grade dysplasia is occasionally reported and may be associated with invasive carcinoma.

Intraluminal neoplastic components of these precursors were classified into two patterns based on the density and distribution of capillaries in their stalks or stroma [11,12].

1. ***Regular and dense capillary pattern***

**Figure 4:**
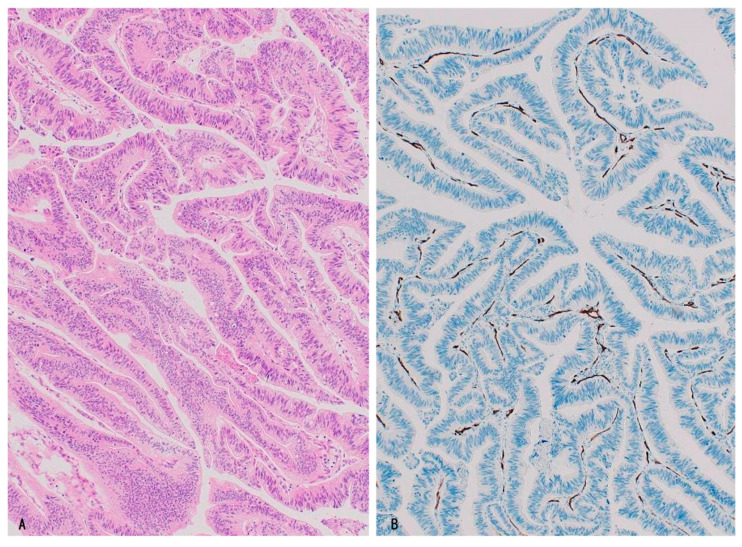
CD34-positive capillaries are regularly found in the fine stalks of papillary neoplasms. Intraductal papillary neoplasm of bile duct (IPNB) (type 1) of intestinal subtype. (**A**), HE; and (**B**), CD34 immunostaining.

About half of IPNB, one-third of ICPNs, and all cases of PGA presented this distribution pattern. Dotted capillaries were regularly and/or rather densely found in all (or almost all) fine stalks or narrow stroma of intraluminal neoplastic components, and these capillaries underlined the neoplastic epithelial layer [11,12] (Figure 4A,B). These neoplastic epithelia and underlying capillaries were continuous with neoplastic epithelia and underlying capillaries in the stalk and stroma of intraluminal neoplasms, respectively. The intraluminal neoplastic component of this capillary pattern showed histological features of type 1 IPNB and ICPN (Table 3) [11,12].

Interestingly, there was little fibrosis in these thin stalks and stroma of the intraluminal components; thus, these capillaries were different from neoangiogenic vessels, a component of wound healing-like reactions with fibrotic stroma, characterizing invasive carcinomas [1,5]. It is conceivable that dense and/or regular capillaries in the fine stalks or stroma might be accompanied by intraluminally proliferating neoplastic epithelia and that these capillaries might have sprouted from capillaries underlining the surrounding flat or micropapillary neoplastic epithelia. Such extension and proliferation of capillaries in intraluminal neoplastic components may be induced by proangiogenic factors such as VEGF expressed in the neoplastic biliary epithelia [3].

A similar vascular proliferative process associated with the expression of proangiogenic factors on biliary epithelia was also reported to occur in PVP of non-neoplastic cholangiopathies, such as primary biliary cholangitis (PBC) and bile duct ligation animal models [16]. These capillaries in the stalks or stroma may be a component of lamina propria of these proliferated intraluminal non-invasive neoplastic components, thus differing from “neo-angiogenesis” of fibrous TME with wound healing-like reaction characterizing invasive carcinoma [1,5] and thereby suggesting that these PCP-like capillaries may be used by intraluminal neoplasms as co-opted vessels.

Interestingly, neoplastic epithelia and underlying PCP of these flat or micropapillary lesions were also continuous with surrounding non-neoplastic biliary epithelia and underlining PCP of the large bile duct and gallbladder [9], implying that these neoplastic epithelia replaced the surrounding non-neoplastic mucosa and used their pre-existing underlining PCP as co-opted vessels, as in high-grade BilIN.

2. ***Sparse capillary pattern***

**Figure 5:**
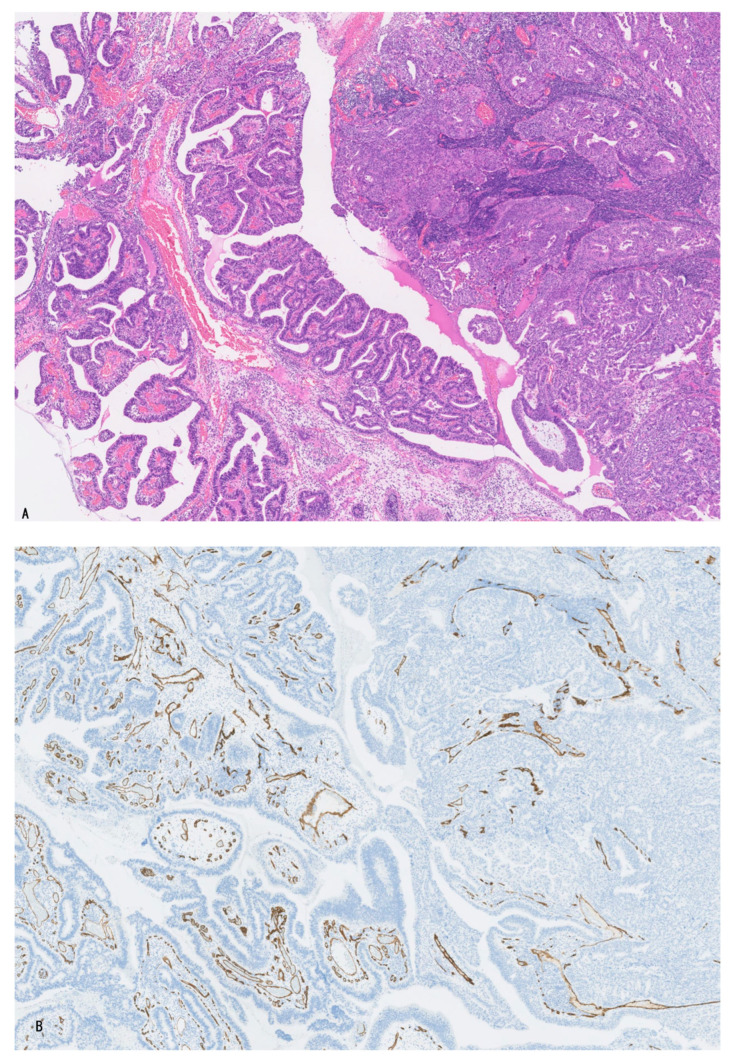
Capillaries are sparse in the intraluminal component, showing solid or cribriform growth (**right** to **center**), while regular and dotted capillaries are found in the stalks of papillary neoplasms in other areas (**left**) in the same case. Intraductal papillary neoplasm of bile duct (IPNB) (type 2) of intestinal subtype with foci of type 1 IPNB areas. (**A**), HE; and (**B**), CD34 immunostaining.

In the remaining half of IPNBs and two-thirds of ICPNs, capillaries were sparse in the intraluminal neoplastic components [10,11] and were absent or barely identifiable in some regions or areas (Figure 5A,B). These intraluminal epithelial neoplastic components showed histological features of type 2 IPNB and ICPN (Table 3) [8,9,20]. One possible explanation for the development of such less-capillarized lesions is that the growth speed of neoplastic epithelial cells might have been much faster and that PCP-like capillaries could not catch up with the speed of neoplastic growth. Another possibility is that neoplastic growth of type 2 IPNB and ICPN does not require an oxygenated capillary blood supply but may rely on anaerobic metabolism for their energy acquisition.

Interestingly, type 2 IPNB or ICPN with sparse capillaries not infrequently contained variable-sized neoplastic areas or components with regular and dense capillaries and type 1 histology (Figure 5A,B), suggesting that type 1 IPNB and ICPN with dense capillaries might have transitioned to type 2 IPNB and ICPN with sparse capillaries [11,12]. Therefore, such a sparse capillary pattern may have developed along with overt malignant transformation of neoplastic epithelial changes in type 1 IPNB and ICPN. Dotted capillaries were regularly distributed in the stalks and intervening stroma in all the PGAs. PGA with invasive carcinoma is not discussed here because of the absence of such cases in our cohort.

3. ***Vessel co-option of and sparse capillaries in intraluminal components of IPNB and ICPN***

In type 1 IPNB and ICPN, neoplastic components are supplied by PCP-like capillaries, and an oxygenated capillary blood supply may be important and mandatory for their growth (Table 2 and Table 3) as in high-grade BilIN. In contrast, in intraluminal neoplastic components of the remaining type 2 IPNB and ICPN with sparse capillaries, oxygenated capillary blood supply might have been markedly reduced, resulting in hypoxia in these areas. Therefore, hypovascularization or hypoxia may be mandatory or beneficial for the growth of type 2 neoplastic epithelia, as seen in LD-iCCA, p/dCCA, and PDAC [4,5,11,12].

While LD-iCCA, p/dCCA, and PDAC are associated with fibrotic and hypoxic stroma with CAFs [5], type 2 intraluminal components of type 2 IPNB or ICPN are not associated with evident fibrosis, thus differing from neo-angiogenesis related to wound-like TME of invasive carcinoma of CCAs and PDAC. It remains to be clarified whether and how such malignant and aggressive feature could also be acquired and maintained in type 2 intraluminal neoplasms of IPNB and ICPN under hypoxic conditions but without fibrous stroma, particularly without the participation of CAFs.

Our previous studies showed that type 2 IPNB and ICPN present more aggressive pathological features, including stromal invasion and worse clinical behaviors, than type 1 IPNB and ICPN [11,20,21]. Thus, the occurrence of type 2 lesions with sparse capillaries in type 1 IPNB and ICPN may indicate the acquisition of biological features equivalent to stromal invasion of the bile duct and gallbladder wall in these cases. In several cases, type 2 intraluminal lesions were focally continuous with invasive lesions in the walls, and there were no confirmative findings that the intraluminal components of almost all cases of type 2 IPNB and ICPN are invasive [9,21]. More confirmatory studies are necessary to elucidate the relationship between type 2 intraluminal lesions and stromal invasion.

#### 2.2.3. Neoplastic Growth in the Walls of the Biliary Tract (Large Bile Ducts and Gallbladder) Associated with High-Grade BilIN, IPNB, and ICPN Referring to Microvasculatures

In the walls of the biliary tract associated with these precursors, two forms of neoplastic glands (glandular involvement by the precursors and invasive carcinoma derived from the precursors) are recognizable (Table 4) [11,12], and they are usually admixed in the walls of the cancerous biliary tract associated with these precursors.

##### Intraepithelial Involvement of Non-Neoplastic Glands

**Figure 6:**
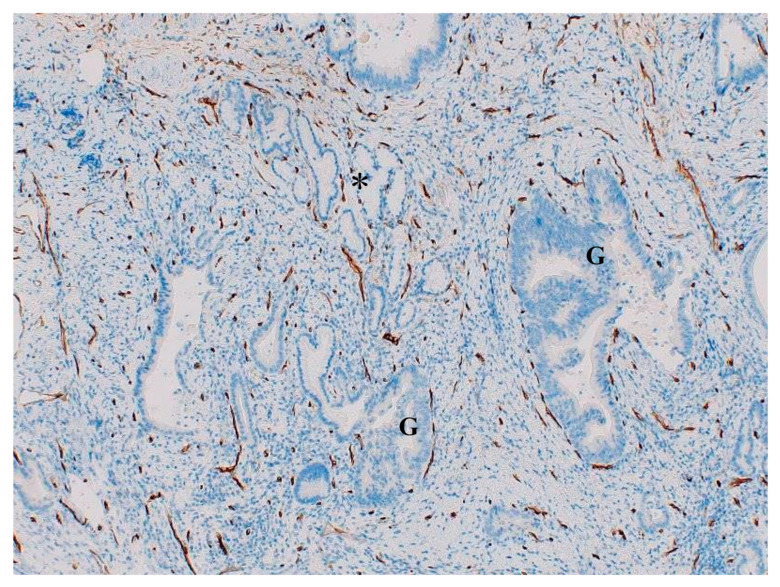
Atypical glands (G) surrounded by C34-positive dotted capillaries are located near the non-neoplastic peribiliary glands (*) of the cancerous bile duct of extrahepatic cholangiocarcinoma. CD34 immunostaining.

The peribiliary glands and their conduits in the large bile duct wall, RAS, and other non-neoplastic glands of the gallbladder wall are frequently involved intraepithelially by the neoplastic epithelium of (i) high-grade BilIN and IPNB and (ii) high-grade BilIN and ICPN as seen in the mucosa surrounding these precursors (see above) [9,11,12]. In such cases, the original PCP surrounding these non-neoplastic glands remained as such, so the involved glands in the wall were surrounded by PCP-like capillaries (Figure 6), which is different from invasive carcinoma [11]. These PCP-like capillaries around the involved glands can be regarded as co-opted vessels [11] because in vessel co-option, a growing tumor preserves the vascular scaffold of the surrounding non-neoplastic tissue and uses them as supplying vessels [1,22].

##### Invasive Carcinoma

Foci of atypical neoplastic glands presenting tubular, cord-like, or micropapillary structure discontinuous with neoplastic epithelia of intramucosal or intraluminal preinvasive neoplasms, and not surrounded by dotted capillaries, were found in the walls of the biliary tract associated with high-grade BilIN, IPNB, and ICPN. They were variably differentiated and were not infrequently associated with desmoplastic reaction [5,11,12]. These glands are considered invasive carcinomas. They were embedded in fibrous stroma, in which small numbers of arteries, veins, and capillaries were found and were not oriented to these neoplastic glands (Figure 2A,B). These vessels are regarded as newly formed blood vessels (neo-angiogenesis) in fibrous stroma [5,11,12].

##### Progression of Precursors into Invasive Carcinoma

In cases with precursors in which invasive carcinoma is only focal, such invasive carcinomas are usually found around or adjacent to the precursors in the mucosa or non-invasive neoplastic glands in the wall of the biliary tracts, suggesting a close or causal relationship between the precursors and invasive carcinoma [9]. Regarding the processes of the development of invasive carcinoma from these precursors, the following are possible: neoplastic glands without PCP-like capillaries (invasive carcinoma) newly develop, proliferate and become predominant. Otherwise, precursors or non-invasive neoplastic glands gradually lose the surrounding capillaries along with malignant transformation and eventually transform into neoplastic glands without surrounding capillaries (invasive carcinoma). The activation of genes such as *hypoxia-inducible factor-1α (HIF-1α)* and *Brahma related gene 1 (BRG1)* and the participation of CAFs in fibrotic and hypoxic tissues may be involved in the development of such tumor environments and in the acquisition of aggressive and resilient invasive CCA, as is speculated to occur in PDAC [5].

##### Significance

The response to chemotherapy may differ between neoplastic glands with surrounding capillaries (vessel co-option) and those without (neoangiogenesis) [1,5,22] because their blood supply is different. Due to the nature of the mode of vascularization, neoplastic glands that rely on vessel co-option, which are found in the mucosa (high-grade BilIN, IPNB, and ICPN) and in the walls of the biliary tract (intraepithelial glandular involvement by these precursors), may be resistant to anti-angiogenic therapies that are routinely used in clinical settings for different cancer types [1,22,23]. Recently, intraductal pancreatic cancer was reportedly less responsive to neoadjuvant chemotherapy than cancer in the stroma [23], possibly due to the different vascular supply as speculated in the vascular supply in neoplastic glands with vessel co-option and those with neoangiogenesis [1,23].

#### 2.2.4. Practical Application of Neoplastic Patterns of Microvascualtures in Histopathologic Diagnosis of LD-iCCA and eCCA and GBC

As described above, neoplastic glands in the walls of the biliary tract with precursors were classified as non-invasive neoplasms (glandular involvement) and invasive carcinomas, and these two types of neoplastic glands are usually admixed in the walls of the biliary tract affected by high-grade BilIN, IPNB, and ICPN. For practical diagnostic procedures including T-categorization of TNM classification of biliary tract neoplasms [24], differentiation of these two forms of neoplastic glands is important. Particularly, glandular involvement should not be counted as invasion but can instead be called “pseudo-invasion” [11,12].

##### Differentiation of Glandular Involvement from Invasion

Glandular involvement is easily recognizable in cases in which only such neoplastic glands are found, or such glands are predominant in the walls of the biliary tract. However, in other cases in which invasive carcinoma is predominant or variably found, differentiation of glandular involvement from invasive carcinoma is not easy or subjective [25,26].

Differentiation of these two glands is an age-old question and is not easy using Hematoxylin–Eosin (HE) staining when both are well-differentiated. To date, several pathologic approaches have been applied to differentiate such non-invasive glands from invasive carcinomas (Table 5) [25,26]. We have added a differentiation approach using endothelial immunostaining of capillaries around the neoplastic glands [11,12]. In detail, the presence of surrounding PCP-like capillaries around the neoplastic glands favors non-invasive neoplasms, while their absence favors invasive carcinomas [11,12]. Neoplastic glands with dotted capillaries are infrequently and focally recognizable deep in the walls of the biliary tract [11,12], and they are usually found near or admixed with non-neoplastic peribiliary glands entrapped in the bile duct wall and periductal tissue and also near or admixed with non-neoplastic glands such as RAS in the gallbladder (Figure 6), suggesting that these non-invasive neoplastic glands may correspond to the peribiliary glands and their conduits and RAS involved intraepithelially by high-grade BilIN, IPNB, or ICPN, which remained in the cancerous bile ducts or gallbladder.

##### T-Category in TNM Classification of LD-iCCA and eCCA and GBC Referring to Glandular Involvement

Our recent study showed that the T-category was considerably changed before and after endothelial immunostaining used for the differentiation of neoplastic glands with (pseudolesion) and without PCP-like capillaries (invasion) [11]. When the T category of high-grade BilIN and ICPN based on this new approach was compared with that based on a histological evaluation, 8 of 38 cases of high-grade BilIN and 13 of 25 cases of ICPN were downstaged after obtaining information on PCP-like capillaries around neoplastic glands. In particular, 6 cases of high-grade BilIN and 12 cases of ICPN belonging to intramucosal (pT1a) and invasive carcinomas (pT1b-3) were re-staged as pTis. The cases staged as pTis based on the information on PCP-like capillaries around neoplastic glands were all alive and showed a favorable postoperative overall survival compared with those of pT1b-3. Therefore, immunostaining of PCP-like capillaries is strongly recommended [11,12].

## 3. Invasion of iCCA into the Hepatic Parenchyma Referring to Vessel Co-Option and Pre-Existing Sinusoids

### 3.1. Non-Neoplastic Hepatic Vascular Pattern and Neoplastic Vascular Pattern in HCC and Metastatic Carcinoma

**Figure 7:**
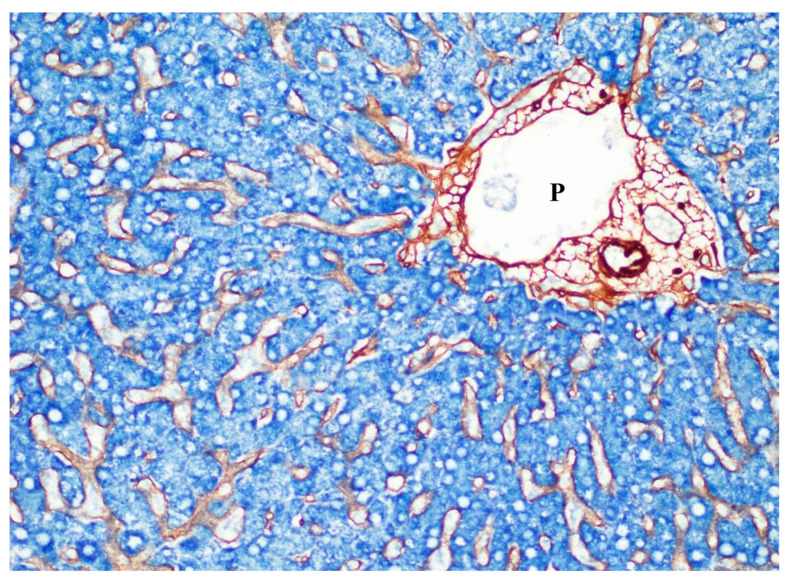
Hepatocytes (blue) arranged in cords are lined by sinusoidal cells (brown) forming hepatocyte–sinusoidal alignment. P, portal vein. Normal liver. Double immunostaining of HepParI (blue) and collagen type IV (brown).

Hepatic lobules, known to be a basic component of the hepatic parenchyma and another highly vascularized region, are dually supplied by terminal hepatic arteries and portal vein branches, and irrigated blood through hepatic lobules flows into the central vein and then into the hepatic veins [14]. The hepatic lobules are based on the cord-like arrangement of hepatocytes along the sinusoids (hepatocyte–sinusoid alignment) (Figure 7), and this alignment is the basic function–structure relationship of the hepatic lobules with a dense vascular network (Table 1) [1,14]. This alignment allows for close interaction between hepatocytes, endothelial cells, and cells in the perivascular space of Disse. In addition, sinusoids are highly specialized, with fenestrated endothelial cells and an incomplete basement membrane [1,14]. These morphological characteristics support the functional units of the hepatic lobules by facilitating the interaction between hepatocytes and sinusoid blood.

During regeneration of the liver after injury, the vascular backbone of the hepatocyte–sinusoid alignment remains stable, and sinusoidal angiocrine factors, as seen in liver organogenesis [27], may instruct new hepatocytes to replace old ones [1,28,29]. This “friendly” growth pattern resembles one of the histopathological growth patterns of liver metastases and HCC [1,6,14,15]. There have been several reports that when HCC and metastatic carcinoma grow against the hepatic parenchyma, carcinoma cells grow by replacing the hepatocytes with reserved sinusoid vessels [1,10,15]. Indeed, in tumors in the liver with such a ‘replacement’ growth pattern, cancer cells co-opt sinusoidal blood vessels by replacing hepatocytes [1]. In this way, a ‘cancer cell–sinusoid alignment’ occurs, and the tumors grow without angiogenesis or desmoplastic reaction [10,15,30,31,32,33].

### 3.2. Microvascular Pattern in iCCA with Correlation to Growth Pattern

We reviewed vessel co-option and hepatocyte–sinusoid alignment referring to the growth pattern of iCCA at the interface between the tumor and adjacent background liver and their relationship to histologic subtypes of iCCA.

iCCA is classified into SD-iCCA and LD-iCCA [8,9,10,11]. LD-iCCA shows invasive characteristics with desmoplastic stromal reactions and preferentially involved large portal tracts. In contrast, SD-iCCA comprises cuboidal to low columnar tumor cells and preferentially develops in the hepatic parenchyma. While LD-iCCA invades the bile duct wall and periductal and portal connective tissue as a natural progression, it also invades the hepatic parenchyma. SD-iCCA also invades the hepatic parenchyma [9,10].

#### 3.2.1. Three Growth Patterns of iCCA Against the Hepatic Parenchyma

Three growth patterns have been recognized in iCCA (Table 6) [1,10], as reported in metastatic carcinomas growing against the hepatic parenchyma [7]. **Replacing growth type** is defined by the direct and continuous growth of tumor cells into the hepatocytic cords with reserved sinusoids, with non-neoplastic hepatocytic cords continuously replaced by tumor cells. There is no fibrous capsule between the tumor and the hepatic parenchyma. **Pushing growth type** is a growth pattern in which tumor cells compress and push the adjacent hepatocytic cords without a rim of desmoplastic pseudocapsule. The hepatic cords surrounding the tumors attach to the adjacent tumor directly, and none of the hepatocytes are replaced by tumor cells. **Desmoplastic growth type** is characterized by a desmoplastic reaction surrounding the tumor nodule (fibrous capsule), which separates the tumor cells from the surrounding hepatic parenchyma. No direct contact between the tumor cells and hepatocytes is seen. This type may correspond to the fibrotic TME of invasive carcinoma with a wound-healing-like reaction [1].

#### 3.2.2. Replacing Growth Pattern and Vessel Co-Option

**Figure 8:**
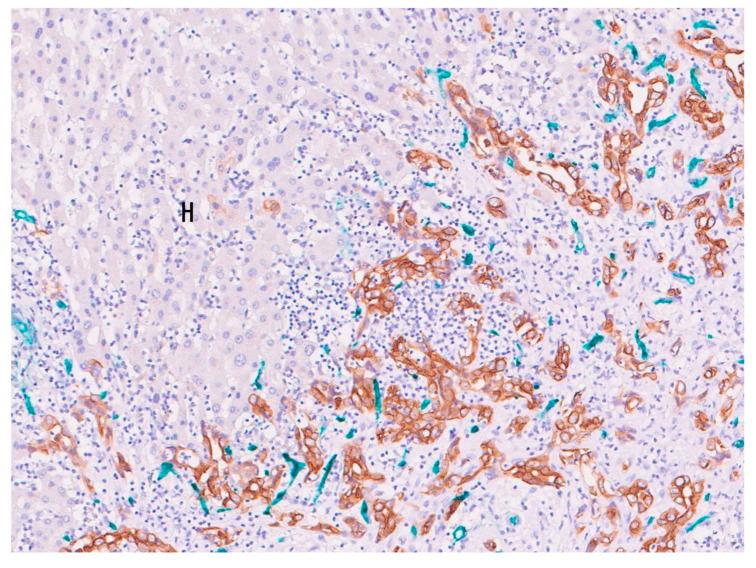
Carcinoma cells (brown) are in direct contact with non-neoplastic hepatocytes (hematoxylin-colored), presenting the replacement of hepatocytes by carcinoma cells. These carcinoma cells are surrounded by blue-colored sinusoidal cells (capillarized sinusoidal cells), suggesting vessel co-option. Sinusoidal cells that are not capillarized in the hepatic lobules are not stained with CD34. Replacement growth of cholangiocellular carcinoma. H, non-neoplastic hepatocyte. Hematoxylin and double immunostaining of CD34 (blue) and CK19 (brown).

Among the three growth types, the replacing growth type used the hepatic sinusoid as co-option vessels. That is, tumor cells continuously replace the pre-existing hepatocytes in the hepatic cord, but sinusoid-like vessels are retained around the cords incorporated by tumor cells. Along with the progression of replacing growth, the co-opted hepatic sinusoids extend into the tumor, and as a result, the intact portal tracts originally located in the parenchyma are also incorporated in the tumor. Nearly intact portal tracts are often embedded within tumor nodules [10], and the overall original hepatic lobule structure and portal tracts are preserved, indicating that pre-existing blood flow is entering the tumor (Table 1) (Figure 8) and thereby implying that the carcinoma cells of iCCA with the replacing growth use the pre-existing sinusoid as blood-supplying vessels (vessel co-option) [1,10].

When tumor cells replace the normal epithelia, a “tumor cell-sinusoid alignment” develops, and the tumors grow in the hepatic lobules without neo-angiogenesis or desmoplastic reaction [10]. The replacing growth pattern is vascularized mainly via vessel co-option and results in a poor response to anti-angiogenic therapy [1,10,22,23]. SD-iCCA with co-option of liver sinusoids may also be related to resistance to immunotherapy because sinusoidal endothelial cells contribute to the immunotolerogenic nature of the liver [34]. Therefore, SD-iCCA supplied by co-option vessels may be resistant to anti-angiogenic therapies routinely used in clinical settings for different cancer types [22,23]. When cancer cells of SD-iCCA are able to grow along sinusoidal blood vessels and preserve the specialized perisinusoidal environment, they probably acquire multiple resistance mechanisms to angiogenesis inhibitors, chemotherapy and immunotherapy [34].

The process of replacement growth of a liver tumor clearly resembles liver organogenesis and regeneration from a morphological point of view [27]. Hypothetically, similar biological mechanisms drive these processes.

In the remaining pushing and desmoplastic growth types, such a “tumor cell–sinusoid alignment” were not recognized and thus they did not use the pre-existing hepatic sinuosids as co-option vessels (Table 6). Particularly, the desmoplastic type may be supplied by newly formed blood vessels in the tumor fibrous microenviroment [1,2,3,4,5].

#### 3.2.3. Histologies of Subtypes of iCCA Showing Three Growth Pattern

Two subtypes of SD-iCCA have been proposed: cholangiolocellular carcinoma (CLC) and iCCA with ductal plate malformation pattern (DPM). While the CLC subtype is similar to reactive bile ductules and is not infrequently mixed with SD-iCCA (SD-iCCA-CLC) [10,35], the DPM subtype is characterized by irregular lumens of varying dilations and shapes, occasionally with microcystic dilation [34].

Li et al. showed that most cases of the DPM, CLC, and SD-iCCA-CLC subtypes adopted the replacing growth pattern [10]. In contrast, the desmoplastic growth pattern was more preferably found in LD-iCCA and pure SD-iCCA, whereas the predominant pushing growth pattern was rare. These data suggest that SD-iCCA with features similar to ductular, progenitor, or stem cells shows replacing growth more frequently than SD-iCCA with features of LD-iCCA. Replacing growth is also a frequent finding in HCC, particularly smaller-size lesions, implying that CCA showing such features may share biological and phenotypic features with HCC and stem cells [15].

However, the replacing growth pattern may also be related to the stages of progressive growth in iCCA [10], where the replacing growth pattern was frequent smaller tumor sizes and lower stages [10]. An increase in tumor size was negatively correlated with the promotion of replacing growth pattern. Furthermore, the replacing growth pattern showed better prognostic trends than the desmoplastic and pushing growth patterns [10], supporting this speculation. However, in colorectal liver metastasis cases, van Dam et al. reported that overall survival was significantly superior in the desmoplastic growth pattern subgroup compared with the replacement or pushing growth pattern subgroup [7], inconsistent with the SD-iCCA cases, which were composed of smaller cases and well differentiated cases [10]. The stage and differentiation of carcinoma and other factors may be responsible for this discrepancy between primary SD-iCCA and metastatic colorectal carcinoma.

#### 3.2.4. Microvasculatures of Precursors of SD-iCCA

Since there are no established precursors in SD-iCCA, alterations of PCP-like capillaries around the small ducts and bile ducts and their unique microvasculatures as found in the precursors of LD-iCCA, p/dCCA and GBC remain unclear.

## 4. Conclusions

Microvasculature of biliary tract neoplasms were reviewed with reference to vessel co-option and pre-existing vessels and to two hypervascularized anatomical regions of the hepatobiliary system: (i) large bile ducts and the gallbladder and (ii) hepatic lobules. In these two regions, biliary epithelia–PCP and hepatocyte-sinusoid alignments present a unique structure–function relationship. High-grade BilIN, PGA, and about half of IPNB and one-third of ICPN with non-complicated structure were underlined by dense and regular PCP-like capillaries and used as co-opted vessels. In contrast, LD-iCCA and GBC were embedded in fibrous stroma containing neo-angiogenic vessels with a wound healing-like reaction. Interestingly, in about half of IPNB cases and two-thirds of ICPN cases with complicated growth, the intraluminal neoplastic components were associated with sparse capillaries and almost no fibrosis, differing from neoangiogenesis or vessel co-option. iCCA shows replacing growth into the hepatic lobules used sinusoids as co-opted vessels. Neoplasms vascularized via vessel co-option may exhibit different responses to anti-angiogenic therapy toward neoplasms vascularized via neo-angiogenic vessels. Visualization of PCP and hepatic sinusoids could be a new pathological tool for the evaluation of malignant progression and vascular supply in CCA and its precursors.

## Figures and Tables

**Table 1:** Highly vascularized regions in the hepatobiliary system.

Anatomical Location	Intrahepatic Large and Extrahepatic Bile Duct and Gallbladder	Hepatic Lobules
Structural units	Biliary lining epithelia underlined by dense and regular peribiliary capillary plexus (PCP) (biliary epithelia–PCP alignment) PCP is composed of fenestrated endothelium with a diaphragm and extreme cytoplasmic attenuation	Hepatocytic cords lined by sinusoidal blood vessels (hepatocyte–sinusoid alignment) Sinusoids are highly specialized with fenestrated endothelial cells and an incomplete basement membrane
Supplying from	Hepatic arterial branches	Hepatic arterial and portal vein branches
Draining into	Portal vein and sinusoids	Central vein
Function	Supporting the secretory and absorptive function of the biliary epithelia into bile, and from bile, respectively	Supporting the transport of constituents from sinusoid blood to hepatocytes and secretion from hepatocytes into sinusoid blood

**Table 2:** Microvasculature of neoplasms arising in intrahepatic large and extrahepatic bile ducts and gallbladder.

Vessels	Neoplasms	Blood Supply
Vessel co-option of pre-existing PCP	High grade BilIN IPNB type 1 ICPN type 1 PGA	Hypervascular Hypervascular Hypervascular Hypervascular
Sparse capillaries	IPNB type 2 ICPN type 2	Hypovascular Hypovascular
Neoangiogenesis as a component of TME	Invasive CCA and GBC Invasive carcionomas associated with precursors	Hypovascular Hypovascular

PCP, peribiliary capillary plexus; BilIN, biliary intraepithelial neoplasm; IPNB, intraductal papillary neoplasm of bile duct; TME, tumor microenvironment; CCA, cholangiocarcinoma; PGA, pyloric gland adenoma; GBC, gallbladder carcinoma.

**Table 3:** Biliary tract neoplasms referring to microvasculature: PCP and other vessels.

Neoplasm	Surrounding Capillaries	Fibrous Stroma (TME) with Neo-Angiogenesis	Significance of Microvasculatures
High-grade BilIN	Regular and dense	Absent	Pre-existing PCP is used as co-opted vessels
Associated invasive carcinoma	Absent	Present	Neoangiogenesis with fibrous stroma (TME)
IPNB Type 1	Regular and dense	Absent	Pre-existing PCP is used as co-opted vessels
Type 2	Sparse	Absent	Poor vascularity and no neoangiogenesis
Associated invasive carcinoma	Absent	Present	Neoangiogenesis with fibrous stroma (TME)
ICPN Type 1	Regular and dense	Absent	Pre-existing PCP is used as co-opted vessels
Type 2	Sparse	Absent	Poor vascularity and no eoangiogenesis
Associated invasive carcinoma	Absent	Present	Neoangiogenesis with fibrous stroma (TME)
LD-iCCA, eCCA	Absent	Present	Neoangiogenesis with fibrous stroma (TME)
GBC	Absent	Present	Neoangiogenesis with fibrous stroma (TME)

PCP, peribiliary capillary plexus; BilIN, biliary intraepithelial neoplasm; IPNB, intraductal papillary neoplasm of the bile duct; ICPN, intracholecystic papillary neoplasms; TME, tumor microenvironment; LD-iCCA, large duct type intrahepatic cholangiocarcinoma; eCCA, extrahepatic cholangiocarcinoma; GBC, gallbladder carcinoma.

**Table 4:** Neoplastic glands in the walls of large bile ducts and gallbladder associated with precursors (high-grade BilIN, IPNB, and ICPN).

	Neoplastic Glands Surrounded by Regular and Dense Capillaries	Neoplastic Glands Not Surrounded by Capillaries
Pathology	• Occasionally found • Close and continuous to non-neoplastic peribiliary glands and conduits • Well differentiation	• Majority of neoplastic glands • Frequently associated with desmoplastic reaction (neoangiogenesis) • Variable differentiation
Significance	• Peribiliary glands and RAS involved intraepithelially by precursors (pseudoinvasion)	Invasive carcinoma itself
T-category	pTis	>pT1a depending on the location of neoplasms

BilIN, biliary intraepithelial neoplasm; IPNB, intraductal papillary neoplasm of bile duct; ICPN, intracholecystic papillary neoplasm; Precursors, high-grade BilIN, IPNB and ICPN.

**Table 5:** Histopathologic features indicative of preinvasive neoplasm and those indicative of invasive carcinoma in the bile duct and gallbladder wall.

	Preinvasive Neoplastic Glands *	Invasive Neoplastic Glands **
Histologic differentiation	Well-differentiated	Well- to poorly differentiated
① Neoplastic tubules continuously penetrating from intraluminal neoplasm into the walls	Could be present	Absent
② Mixture or adjacent localization of neoplastic glands within or to non-neoplastic glands such peribiliary glands and RAS	Could be present	Absent
③ Association with active desmoplastic reaction	Absent	Not infrequent
④ Presence of inspissated bile in long dilated spaces	Could be present	Unrelated
⑤ Long tubular often dilated structures extending through the intermuscular connective tissue	Could be present	Absent
⑥ Similar cytological and immunophenotypic features to intraluminal components	Similar to intraluminal preinvasive components	Could be different from intraluminal preinvasive components
⑦ Coexistence of neoplastic and non-neoplastic epithelia in the same glands	Could be present	Absent
⑧ Surrounding dotted capillaries	Present	Absent
⑨ Small or medium-sized atypical glands infiltrating into the small muscular bundles or intermuscular connective tissue	Absent	Could be present
⑩ Perineural and/or intravascular invasion of neoplastic glands	Absent	Could be present
⑪ Exposure of neoplastic cells on the serosa	Absent	Could be present

①~⑦ favor preinvasive neoplastic glands, while ⑧~⑪ favor invasive glands; *, peribiliary glands and their conduits and RAS intraepithelially involved in neoplastic epithelia of precursors; **, invasive carcinoma.

**Table 6:** Growth patterns of invasion of intrahepatic cholangiocarcinoma into hepatic lobules.

	Replacing Growth	Pushing Growth	Desmoplastic Growth
Direct contact of tumor cells with non-neoplastic hepatocytes in hepatocytic cords	+	+	-
Vessel co-option of sinusoids	+	-	-
Original hepatic lobular pattern	Reserved	Not found	Not found
Entrapment of intact portal tracts	+	-	-
Desmoplastic capsule	-	-	+
